# Dissolution of Intact, Divided and Crushed Circadin Tablets: Prolonged *vs*. Immediate Release of Melatonin

**DOI:** 10.3390/pharmaceutics8010002

**Published:** 2016-01-07

**Authors:** Hui Ming Chua, Nathalie Hauet Richer, Magda Swedrowska, Stephen Ingham, Stephen Tomlin, Ben Forbes

**Affiliations:** 1 Institute of Pharmaceutical Science, King’s College London, King’s Health Partners, London SE1 9NH, UK; chua81@gmail.com (H.M.C.); nataloldigo@gmail.com (N.H.R.); magda.swedrowska@kcl.ac.uk (M.S.); steve.ingham@kcl.ac.uk (S.I.); 2 Pharmacy Department, Evelina London Children’s Hospital, Guy’s & St. Thomas’ NHS Foundation Trust, King’s Health Partners, London, UK; Stephen.tomlin@gstt.nhs.uk

**Keywords:** sleep, insomnia, paediatric, autism, ADHD, quality control, quality assurance, pharmacoenconomics, controlled release

## Abstract

Circadin 2 mg prolonged-release tablet is the only licensed melatonin product available in the UK. Circadin is indicated for patients with primary insomnia aged 55 and over, but is more widely used “off-label” to treat sleep disorders especially in the paediatric population. Children and older people often have difficulty swallowing tablets and dividing the tablet is sometimes required to ease administration. The aim of this study was to measure the release profile of melatonin from Circadin tablets when divided or crushed, and compare this with release from intact tablets. Dissolution testing was also performed for unlicensed melatonin products for comparison. Dissolution tests were performed using the pharmacopoeial paddle apparatus, with melatonin release analyzed by high performance liquid chromatography. Melatonin content, hardness, friability, and disintegration of the products were also evaluated. The prolonged release of melatonin from Circadin tablets was unlike that of any other product tested. When divided into halves, Circadin preserved most of the prolonged-release characteristic (f2 = 58), whereas quarter-cut and crushed tablet had a more immediate melatonin release profile. Circadin is significantly less expensive and should be preferred to unlicensed medicines which are not pharmaceutically equivalent and offer less quality assurance.

## 1. Introduction

Melatonin (5-methoxy-*N*-acetyltryptamine) is a natural hormone produced mainly by the pineal gland in the brain. The secretion of this hormone is influenced by the presence and absence of light, with the plasma level peaking in the middle of the night and gradually reducing towards the morning [1,2]. Abnormal melatonin secretion affects the biological clock and results in disturbance of the physiological functions regulated by the circadian rhythm. Melatonin plays a role in the control of the sleep-wake cycle, hence its use in treating sleeping disorders has been widely studied [3,4,5,6]. Sleep problems are common in the clinical practice, especially in paediatric populations [7] and melatonin is used widely to treat childhood insomnia when behavioural interventions have failed. Although melatonin has a variety of physiological effects apart from altering sleep timing and unproven safety assurance for long term use in paediatrics [8], it has less overt adverse effects compared to other sleep-inducing drugs [6,9,10]. Most of the studies examining the effects of melatonin in children and adolescents report the use in medical conditions such as autism spectrum disorders [11,12,13,14,15,16], attention deficit hyperactivity disorders [17,18,19], and other neurodevelopmental disorders [20,21,22]. In 2008, it was estimated that 5000 children in the UK with sleep disorders were being treated with melatonin [23].

Oral absorption of melatonin is rapid and peak plasma levels are achieved 20 to 60 min following ingestion. The plasma half-life of melatonin is short and it is rapidly cleared by the liver [3,24]. A variety of experimental evidence exists for the efficacy of melatonin in patients with circadian rhythm sleep disorders [3,4,25,26]. A recent meta-analysis of randomized, placebo-controlled trials examining the effects of melatonin for the treatment of primary sleep disorders in adults and children concluded that although the effects were modest, melatonin decreases sleep onset latency, increases total sleep time, and improves overall sleep quality [27]. One study comparing the effect of melatonin in prolonged-release dosage form and immediate-release dosage form concluded that melatonin in the form of immediate-release was most effective for treating delayed sleep onset, whereas prolonged-release melatonin was more useful for sleep maintenance [22].

In the United States, melatonin is not licenced as a medicine by the Food and Drug Administration but is sold in various formulations as a dietary supplement. In contrast, in the UK melatonin is categorized as a medicine by Medicines and Healthcare Products Regulatory Agency and a single product, Circadin (2 mg prolonged-release tablets), has had marketing authorization since 2008 [28]. The initial indication for Circadin, primary insomnia in adults aged 55 years and over, was extended from 3 weeks to 13 weeks in June 2010 following submission of additional data. Circadin is also prescribed widely to treat insomnia in individual paediatric and adolescent patients as an “off-label” use [29]. Prior to the introduction of Circadin in 2008, there were more than 50 melatonin preparations being used in the UK, including tablets, capsules, and oral solutions in different strengths [23,30]. Some of these products were produced by “special manufacturers” in the UK to cater for off-licence demand, some were imported, and some were purchased from online stores by patients or the caregivers of young patients [29]. The MHRA issued drug procurement advice in August 2008 emphasizing that the licensed product, Circadin, should be used wherever possible rather than unlicensed alternatives [31]. However, a variety of unlicensed products are still being used (33 according to Prescription Cost Analysis data in 2015), which is a quality and safety concern. The National Health Service (NHS) UK issued a bulletin in July 2013 outlining guidance for “choosing between melatonin formulations for treatment of sleep disorders” recommending that Circadin should be prescribed as the first choice upon any initiation of melatonin treatment and existing patients on melatonin using unlicensed products should be changed to Circadin after treatment review [32].

The release profile of melatonin from Circadin tablets features an initially faster release (approximately a third of the dose in the first hour) followed by a slower prolonged release for up to eight hours, which is apposite for promoting the induction and maintenance of sleep. Despite the relatively small dimensions of Circadin (white, round, biconvex tablets with a diameter of 8.1 mm and thickness of 3–5 mm), some patients have difficulty swallowing the formulation intact. The elderly, disabled, and young demographic for whom this medication is most frequently prescribed may require Circadin tablets to be divided or crushed to ease administration. Since breaking or crushing the Circadin tablet is inconsistent with product SmPC this would, again, constitute “off-label” use. Breaking the intact tablet would be expected to result in loss of the prolonged-release properties and the alteration of its clinical effect [32]. Hence, the principal aim of this study was to evaluate the melatonin release profile of Circadin when divided or crushed. Alternative melatonin pharmaceutical formulations and healthcare supplements were also evaluated and compared to Circadin in terms of their dissolution profile, cost, and quality.

## 2. Materials and Methods

### 2.1. Materials and Reagents

The reference standard of melatonin (99% purity) was obtained from USP Manufacturer, Beijing, China (Batch Number: HOM3611380105). HPLC grade methanol was purchased from Fisher Scientific, UK, deionized water was supplied by Purelab Ultra, Elga, High Wycombe, UK. A total of seven melatonin products available as solid dosage forms were obtained by local or online purchase (Table 1). Circadin was supplied by Flynn Pharma, Stevenage, UK.

**Table 1:** Solid oral dosage forms containing melatonin used in the study.

Product	Dosage Form/Melatonin Dose	Manufacturer	Cost/Unit (£)	Country	Batch Number
*Circadin* Licensed medicine (UK)	Tablet 2 mg (Prolonged release)	Neurim Pharmaceuticals	0.51	Germany	457028201
*Bio Melatonin* Licensed medicine (Hungary)	Tablet 3 mg	Pharma Nord	2.92	Denmark	1450402
Unlicensed medicine (UK “special”)	Capsule 2 mg	Thame Laboratories	1.44	UK	L1405038
Unlicensed medicine (UK “special”)	Tablet 3 mg	Thame Laboratories	1.50	UK	S64622
Food supplement	Tablet 3 mg	Natrol	0.08	USA	2056049
Food supplement	Capsule 3 mg	Vitasunn	0.05	USA	Not provided
Food supplement	Tablet 3 mg (Prolonged release)	Eurovital	0.13	USA	308354

### 2.2. High-Performance Liquid Chromatography (HPLC) Assay

A Waters 2795 Separations Module HPLC was used, equipped with Waters 2996 Photodiode Array Detector. The column was a Luna^®^ 3 µm C18 (2) 100 Å LC Column 150 × 4.6 mm with TMS endcapping. Data was digitized by Empower pro program (Waters, Elstree, UK) for chromatograms acquisition and integration. The calibration curve constructed by the standard melatonin solution was used to quantify each sample peak obtained. Stock solutions of melatonin (10 µg/mL) were prepared by dissolution of 50 mg of the melatonin reference standard in 200 mL of 75% *v*/*v* methanol, sonicating for 45 min, followed by 25-fold dilution with methanol. Serial dilution was performed from this stock solution to obtain a series of melatonin standard solutions (5, 2.5, 1, 0.5, 0.25, 0.1, 0.05 and 0.025 µg/mL) for construction of the calibration curve.

An isocratic mobile phase consisting of methanol-0.6% (*w*/*v*) aqueous ammonium acetate solution (75:25 *v*/*v*), filtered through 0.45 µm nylon membrane filters (Whatman, Little Chalfont, UK) was used in the HPLC analysis. The flow rate of 0.5 mL/min [33] and pressure limit of 4000 psi was set, with the injection volume of 50 µL. Samples were analysed in duplicate with run time of 7 min. The column temperature was set at 40 °C [34] and the UV detection wavelength used was 228 nm.

### 2.3. Dissolution of the Melatonin Products

The rotating paddle dissolution testing method was used according to pharmacopoeial specifications [34]. The dissolution medium was 900 mL of 0.1 M hydrochloric acid, pH 2, with the temperature thermostated to 37 °C. To initiate the dissolution test, a dosage unit was added to each dissolution vessels. Rotating paddle speed was maintained at 50 revolutions per minute and sampling was performed at specified time points: 1, 3, 5, 10, 15, 30, 45, 60, 120, 180, 240, 300, 360, 420 and 480 min. At each time point, 1.5 mL of aliquot was withdrawn from each vessel via syringe and filtered through a 0.20 µm pore size filter unit before analysis by HPLC method as described above. Each vessel was replenished with 1.5 mL of the dissolution medium immediately after withdrawal of a sample.

Circadin tablets were tested in five different forms, which included intact, halved, quartered, manually crushed, and mechanically crushed, with six replicates for each form. A Safe & Sound Pill Crusher & Cutter (manufactured by Boots, Nottingham, UK) was used to cut the tablet into half and quarter. The same device was used to crush the tablet into fine powder (termed as “mechanically crushed”). To obtain the “manually crushed” powder, the tablet was pressed against a spoon by a spatula to disintegrate the tablet resulting in a powder that was coarser than the powder produced by the tablet crusher device. The mean particle size of the powder obtained by mechanically crushing tablets was finer compared to that obtained by manual crushing, particle diameter of 447 µm compared to 709 µm, respectively. Six other melatonin products (Table 1) were tested in triplicate by the same dissolution method.

### 2.4. Quality Attributes: Melatonin Content, Disintegration, Friability, and Hardness

The melatonin content of each of the melatonin products (*n* = 3) was measured by completely dissolving of individual dosage units in 1 L 0.1 M hydrochloric acid, filtering the resultant solution through a 0.20 µm pore size filter unit and analysing by HPLC.

The immediate-release formulation were tested for disintegration. Apparatus A for normal size tablets and capsules was used according to pharmacopoeial methods [34]. The disintegration test was conducted at 37 °C, for a single dosage unit placed in each of the 6 tubes of the basket (six tablets tested concurrently for each product). After 15 min of testing, the baskets were lifted from the water bath to observe whether the dosage units had disintegrated.

Friability and hardness testing was conducted for the tablet dosage forms using Sotax Friabilator F2 and Tablet Hardness & Compression Tester C50, respectively. For friability, 20 tablets for each of the melatonin products in tablet form were tested in a friabilator using 100 rotations. Loss of powder was determined as a percentage and compared to the pharmacopoeial specification (<1% loss). For hardness, one tablet at a time was placed into the tester and the force (kg) to break the tablet was measured (*n* = 10).

### 2.5. Data Analysis

Then similiarity factor, f2 was used to compare the dissolution profiles obtained [35]. The similarity factor was calculated by the following formula [35] where n was the number of time points, R_t_ referred to the dissolution values of Circadin intact (as the reference) and *T*_t_ was the dissolution values of the test products (Circadin in divided and crushed forms or other comparison products), *w*_t_ was set as equal to one at each time point in this case (an optional weighting factor) [35].

(1) $$f_{2}=50\log_{10}\left\{{\left[1+\frac{1}{n}\overset{n}{\underset{t=1}{\sum }}w_{t}{\left(R_{t}-T_{t}\right)}^{2}\right]}^{-0.5}\times 100\right\}$$

Program DDSolver was used to derive the representative kinetic equation for the release profile of Circadin in different forms [36].

## 3. Results and Discussion

### 3.1. HPLC Optimisation

**Figure 1:**
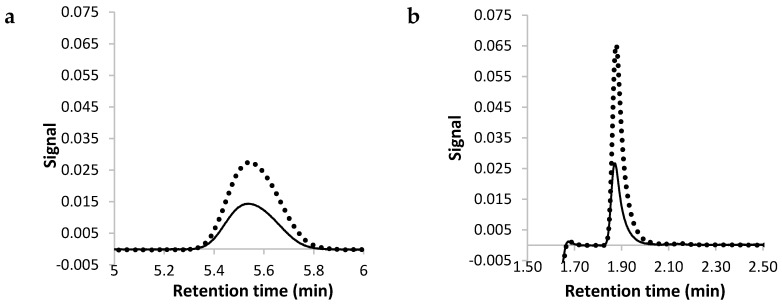
The chromatograms for 1 µg/mL (dotted line) and 0.5 µg/mL (solid line) melatonin using mobile phase containing (**a**) methanol 40%, (**b**) methanol 75%.

**Figure 2:**
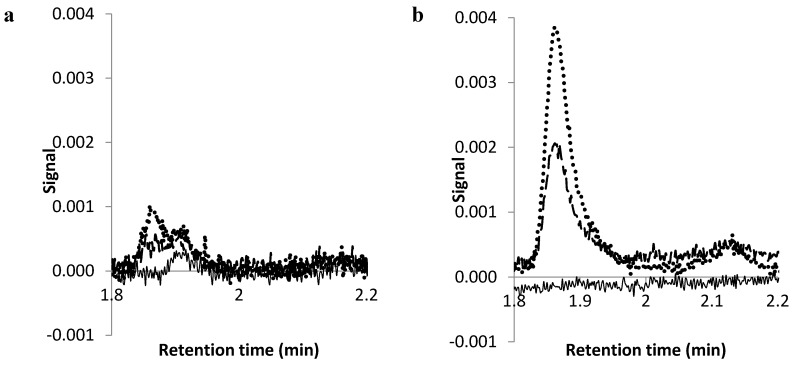
The chromatograms for 0.1 µg/mL (dotted line), 0.05 µg/mL (dashed line) melatonin and baseline 75% methanol (solid line) using UV detection at a wavelength of (**a**) 285 nm (**b**) 228 nm.

The current Pharmacopoeial assay for melatonin using HPLC recommends 400 volume methanol, 600 volume water containing 1 volume of orthophosphoric acid as the mobile phase [34]. The mobile phase was adjusted to use ammonium acetate and 75% methanol as an earlier, sharper peak was obtained and the sensitivity of the analysis was increased (Figure 1). The UV detection wavelength was adapted from 285 to 228 nm as preliminary experiments revealed that the lower wavelength gave higher sensitivity (Figure 2). Using this method, the calibration curve was linear from the 0.025 to 10.0 µg/mL (9 points) with the correlation coefficient (*R* value) of 1.

### 3.2. Dissolution Profiles of Circadin Tablets in Intact and Divided Forms

**Figure 3:**
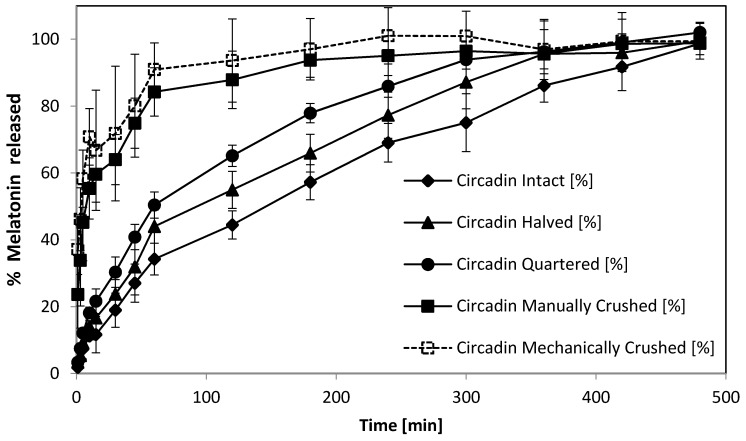
Comparison of dissolution profiles for release of melatonin from Circadin tablets in intact, halved, quartered, and crushed form (data represented mean ± SD, *n* = 6).

The dissolution profiles for Circadin tablets in intact, halved, quartered, manually crushed, and mechanically crushed forms were measured for up to 8 h (Figure 3). The release from intact and halved tablets increased gradually throughout the duration of testing with the dissolution rate for the intact tablets was the most prolonged, followed by the halved form with the quartered form, and finally the crushed forms which resulted in an almost immediate release as soon as the powders were introduced into the dissolution medium. The powders produced using the pill crusher showed quicker release than the manually crushed powders, corresponding to finer particle size providing a larger surface area for dissolution. Both crushed powders released more than 20% of the melatonin content within the first minute of the experiment.

The release of melatonin at one hour (T60) and two hours (T120) for Circadin tablets in the intact and divided forms were recorded in Table 3. The similarity factor, f2 [35] was used to compare the dissolution profile of divided and crushed Circadin tablets with reference to the intact tablet and the derived kinetic models that best fit each dissolution profile were also calculated [36]. When the dissolution profiles of two formulations are identical, f2 value obtained will be equal to 100. When the value is less than 100 but larger than 50, the two formulations are regarded as exhibiting similar release characteristics. This value has been recognized by FDA and EMEA as the official tool to assess the similarity of *in vitro* release characteristics between two formulations in the registration guidelines [37].

**Table 2:** Kinetics of melatonin release from Circadin tablets, intact and after dividing or crushing the tablets (*n* = 6).

Circadin Tablets	Melatonin Release (%) after 60 and 120 min (mean ± SD)		Best Fitting Kinetic Model	f2 *vs*. Intact
	*T*_60_	*T*_120_		
Intact	34.2 ± 4.7	44.5 ± 4.2	Korsmeyer–Peppas Model	Not applicable
			*F* = *F*0 + kKP*t^n 2,3,4^	
			*R*^2^ = 0.9983	
Halved	44.0 ± 5.0	55.0 ± 5.5	Korsmeyer–Peppas Model	58
			*F* = *F*0 + kKP*t^n 2,3,4^	
			*R*^2^ = 0.9943	
Quartered	50.4±3.9	65.1 ± 3.2	Weibull Model	45
			*F* = *F*max*{1-Exp[-(*t*^β^)/α]} ^2,5,6^	
			*R*^2^ = 0.9988	
Manually crushed	84.3 ± 9.2	87.9 ± 8.3	Weibull Model	23
			*F* = *F*max*{1-Exp[-(*t*^β^)/α]} ^2,5,6^	
			*R*^2^ = 0.9914	
Machine crushed	91.0 ± 7.9	93.6 ± 12.4	Weibull Model	19
			*F* = *F*max*{1-Exp[-(*t*^β^)/α]} ^2,5,6^	
			*R*^2^ = 0.9914	

^2^ *F* is the fraction (%) of drug released in time t in all models; ^3^ *F*0 is the initial fraction of the drug in the solution resulting from a burst release; ^4^ kKP is the release constant incorporating structural and geometric characteristics of the drug-dosage form; *n* is the diffusional exponent indicating the drug-release mechanism; ^5^ *F*max is the maximum fraction of the drug released at infinite time; ^6^ α is the scale parameter which defines the time scale of the process; β is the shape parameter which characterizes the curve as either exponential (β = 1; case 1), sigmoid, S-shaped, with upward curvature followed by a turning point (β > 1; case 2), or parabolic, with a higher initial slope and after that consistent with the exponential (β < 1; case 3) [36]

The result of this study showed that dividing Circadin tablets in half did not affect the prolonged-release characteristics, indicated by the f2 value of 58 compared to the Circadin intact tablet (Table 2). When the Circadin tablets were further divided into quarter form or crushed forms, the release characteristics were altered, with all f2 values obtained less than 50 and the prolonged release profile no longer preserved. The kinetic model that best represented the dissolution profile of each form was derived by DDsolver, a program with built-in model library designed to facilitate the dissolution data modelling based on nonlinear optimisation methods [36]. The highest value of coefficient of determination, *R*^2^ was used to assign the most appropriate model for each release profile. For the Circadin tablet in intact and halved form, the release profiles were best represented by Korysmeyer–Peppas model, which describes the drug release mechanism by diffusion from a controlled-release polymeric system [37]. The Weibull model was found to best represent the dissolution profiles of Circadin tablets in quarterly-cut form and crushed forms. The equation derived from this model applies generically to dissolution profiles and lacks kinetic fundament, thus it is not indicative of the drug release mechanism of a pharmaceutical product [37].

Overall, these results (Figure 3, Table 2) confirm, but extend considerably, the limited and incomplete data on the dissolution of intact and divided tablets held on file by Flynn Pharma that several NHS Clinical Commissioning Groups in the UK have used to inform policies on melatonin dispensing [38].

### 3.3. Comparison of Melatonin Release by Circadin and Other Products

**Figure 4:**
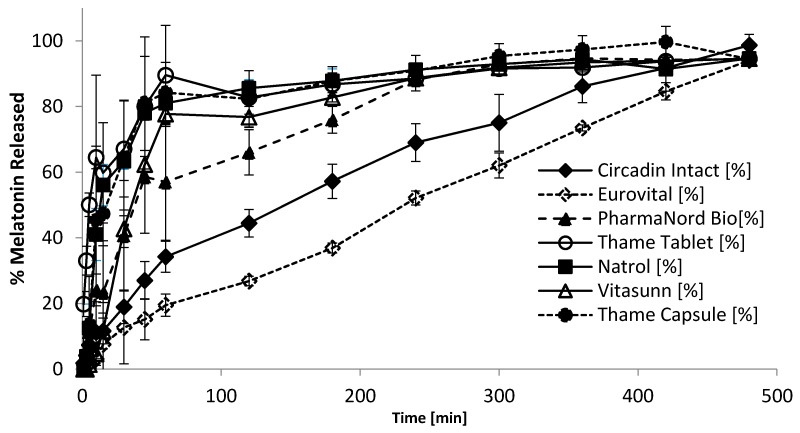
Comparison of dissolution profiles for melatonin tablet and capsule products (Data represent mean ± SD, *n* = 3).

The dissolution profile of Circadin tablets (in intact form) was compared to other unlicensed products (Figure 4). The Circadin dissolution profile was distinct from each of the other products (f2 *vs*. Circadin < 50; Table 4). The melatonin product purchased from Eurovital, USA exhibited sustained-release characteristics as claimed. However, the dissolution rate of this product was markedly slower than the Circadin tablet (Figure 4). The rest of the melatonin products showed immediate release characteristics, with the product manufactured by Natrol, Chatsworth, CA, USA, having the highest rate of dissolution showing more than 50% of drug was released at the first minute of the experiment. Each of the immediate release products liberated >50% of the drug within the first hour of the testing (*T*_60_ > 50%; Table 4).

The dissolution profiles of other melatonin-containing products were compared with those of Circadin when the tablet was divided or crushed (Table 4). Circadin tablets in quarterly-cut form showed similar dissolution profile as the Pharma Nord Bio-Melatonin Tablet (f2 value of 59), an immediate-release dosage form. Circadin in the crushed form (both crushed manually and mechanically) yielded dissolution profiles that were similar to the Thame 3 mg Tablet another immediate-release product, (f2 values of 62 and 52, respectively) when tested under the same condition.

**Table 3:** Kinetics of melatonin release and comparison of dissolution profiles using the f2 similarity factor (>50 indicates similarity) for melatonin tablet and capsule products (*n* = 3).

Melatonin Tablet or Capsule	Release Profile Parameters (%) Mean (± SD)		f2 *vs*. Circadin Intact	f2 *vs*. Circadin Halved	f2 *vs*. Circadin Quartered	f2 *vs*. Circadin Manually Crushed	f2 *vs*. Circadin Machine Crushed
	T60	T120					
Thame 2 mg capsules	84.3 (±9.2)	82.4 (±8.6)	26	30	35	43	34
Thame 3 mg tablets	89.6 (±15.1)	82.8 (±5.1)	22	25	28	64	52
PharmaNord Bio-Melatonin 3 mg tablets	57.0 (±17.8)	66.1 (±6.9)	40	48	59	33	26
Natrol 3 mg tablets	81.1 (±7.9)	85.6 (±6.3)	28	30	35	43	34
Vitasunn 3 mg capsules	77.8 (±15.8)	76.8 (±3.9)	34	41	46	30	24
Eurovital SR 3 mg tablets	19.5 (±3.4)	26.8 (±1.5)	47	37	31	17	14

**Table 4:** Other quality parameters evaluated for all melatonin products.

Product	Label (mg)	Content Analysis (*n* = 3)		Hardness (*n* = 10)	Friability (*n* = 20)	Disintegration (*n* = 6)
		Recovery (mg ± SD)	Deviation (%)	(kg)	(% loss)	(time)
Circadin SR 2mg tablet	2	2.04 ± 0.08	1.99	6.04 ± 0.68	<1%	Not applicable
Thame 2 mg capsules	2	1.83 ± 0.05	8.34	Not applicable	Not applicable	<15 min
Thame 3 mg tablets	3	2.78 ± 0.25	7.34	6.69 ± 0.48	<1%	<15 min
PharmaNord Bio-Melatonin 3 mg tablets	3	2.50 ± 0.19	16.57	2.59 ± 0.66	<1%	<15 min
Natrol 3 mg tablets	3	2.91 ± 0.29	3.05	11.46 ± 1.01	<1%	<15 min
Vitasunn 3 mg capsules	3	2.62 ± 0.34	12.74	Not applicable	Not applicable	<15 min
Eurovital SR 3 mg tablets	3	2.54 ± 0.35	15.34	10.9 ± 1.29	<1%	Not applicable

### 3.4. Product Quality, Packaging, and Labelling

A total of seven melatonin products in solid dosage form obtained from different sources were tested in this study. The results for the testing of melatonin content analysis, hardness, friability and disintegration were recorded in Table 4. Melatonin content measurement revealed that the melatonin recovered from Circadin deviated least from the label strength compared to the unlicensed products, with 2.04 mg of melatonin recovered from 2 mg as claimed by the label. The EU-licensed product, Bio-melatonin 3 mg tablet manufactured by Pharma Nord, Vejle, Denmark had the highest deviation (16.57%) in this analysis. All products passed the other quality control tests (hardness, friability, disintegration).

Only Circadin tablets and a supplement product, Eurovital tablets purchased from USA, claimed to exhibit prolonged-release characteristics. All other products were presumed to have immediate-release formulation, including the two products in capsule form. One of the products evaluated was manufactured by Pharma Nord, Denmark (European Union country) and licensed only in Hungary, with the cost of £2.92 for each tablet recorded as the most expensive product among all products tested. This price was nearly six times higher than the UK-licensed product, Circadin which cost only £0.51 for one tablet. The two unlicensed products manufactured in UK by “special manufacturer” (Thame Laboratories, Ruislip, UK) were the second and third most expensive product in term of the unit cost (£1.50 per tablet and £1.44 per capsule, respectively). Three products being sold as dietary supplements in USA were the least expensive products with the unit cost ranging from £0.05 to £0.13.

The packaging and labelling for each product was evaluated by referring to the “Best Practice Guidance on the Labelling and Packaging of Medicines” by MHRA. Only Circadin conformed to the MHRA requirements. All essential information included medicine name, strength, route of administration, posology, warnings as required by MHRA were displayed with the comprehensive package insert being included with the Circadin tablets, which were packed individually in aluminium strips. The Pharma Nord Bio-melatonin tablets were the only other product packed in blister form and came with comprehensive labelling in the format of a patient information leaflet, as licensed by the authority of European Medicine Agency (EMA).

## 4. Conclusions

The effect of dividing or crushing Circadin tablets on the release of melatonin has been demonstrated and can be used to inform clinical decision-making. The tablets should be used in intact or halved form (when necessary to ease administration) when a prolonged-release characteristic is desired. If divided into quarter or crushed form, the release profile becomes more like that of an immediate release preparation. Circadin is available as a licensed medicine at lower cost than “pharmaceutical special” manufactured alternatives which are not pharmaceutically equivalent. The quality assurance and cost saving benefits of using Circadin impel the use of this medicine over unlicensed or non-medically regulated products.
